# Acute Heart Failure in a Patient with Occult Barlow’s Disease Receiving Bevacizumab

**DOI:** 10.3390/medicina57100998

**Published:** 2021-09-22

**Authors:** Toshihide Izumida, Teruhiko Imamura, Yohei Ueno, Kazuaki Fukahara, Koichiro Kinugawa

**Affiliations:** 1 Second Department of Medicine, University of Toyama, Toyama 930-0194, Japan; m07011ti@jichi.ac.jp (T.I.); fef6ge@gmail.com (Y.U.); kinugawa0422@gmail.com (K.K.); 2 Department of Surgery 1, Faculty of Medicine, University of Toyama, Toyama 930-0194, Japan; fuka@med.u-toyama.ac.jp

**Keywords:** bevacizumab, heart failure, cardiotoxicity

## Abstract

Bevacizumab is a recombinant humanized monoclonal antibody and a key drug for treatment of various types of cancer. Bevacizumab is associated with the occurrence of heart failure, but its risk factors remain unknown. A 55-year-old woman was diagnosed with cervical cancer, which was completely treated by bevacizumab-incorporated chemotherapy. During the 9-month bevacizumab therapy, she suffered from hypertension requiring multiple antihypertensive agents. She was admitted to our hospital due to acute heart failure with afterload mismatch and severe mitral regurgitation. A transesophageal echocardiography showed Barlow’s disease with a degenerated and widely prolapsed mitral valve. She received a scheduled surgical mitral valve repair. Post-operative cause was uneventful, but metastatic dissemination developed later. The existence of mitral valve regurgitation, even when sub-clinical, might be a risk of worsening heart failure during bevacizumab therapy. Careful follow-up at an onco-cardiology clinic is highly encouraged particularly for such a cohort during bevacizumab therapy.

## 1. Introduction

The association between cardiology and oncology, known as an onco-cardiology, has received great concern thus far [1]. Various malignant tumors accompany the cardiovascular diseases. Also, several oncological medications increase the risk of cardiovascular diseases, including systemic hypertension and worsening heart failure [1,2]. For antitumor therapy, it is key to maintain the balance between antitumor effect and cardiovascular toxicity [2].

Bevacizumab is a recombinant humanized monoclonal antibody, targeting vascular endothelial growth factor A, and has recently been put into use for cancer treatment [3]. It has recently been found that bevacizumab increases the risk of hypertension by inhibiting vascular endothelial growth factor-mediated vasodilation, decreasing capillary density, and increasing arterial stiffness [4]. However, detailed analyses including the risk factors for worsening heart failure during the bevacizumab therapy remains unknown.

Here, we experienced a patient with cervical cancer and occult Barlow’s disease, which is defined as excessive mitral leaflet and myxomatous degeneration with mitral leaflet prolapse in addition to generalized billowing [5]. The patient suffered from acute decompensated heart failure and severe degenerative mitral regurgitation, probably triggered by bevacizumab-induced systemic hypertension and afterload mismatch.

## 2. Case Presentation

### 2.1. Before Admission

**Figure 1:**
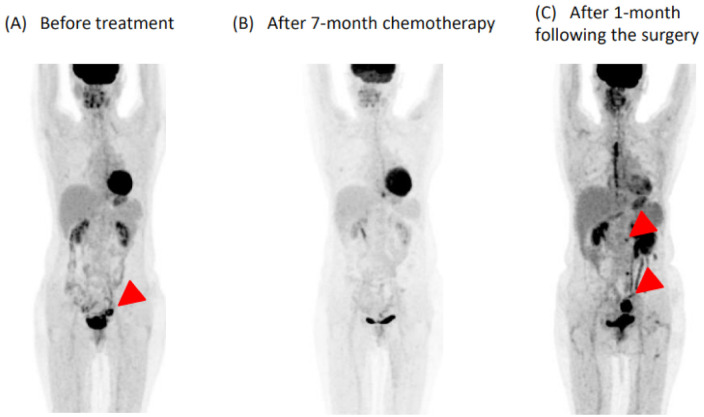
FDG-PET/CT course of cervical cancer (**A**) FDG-PET showed abnormal accumulation in the paracervical ligament (arrow). (**B**) Disappearance of a significant ligament uptake on FDG-PET/CT during BEV therapy. (**C**) FDG-PET showed abnormal accumulation in the membrane of sigmoid colon and para-aortic lymph nodes at 3 months after mitral valve repair (arrows).

A 55-year-old woman was diagnosed stage IIb cervical cancer (Figure 1A). She had never received a medical checkup and her comorbidity history including valvular diseases remained uninvestigated. Her blood pressure was around 110/70 mmHg. The patient was treated with chemoradiation therapy including cisplatin, but the disease progressed further. The patient received cisplatin + paclitaxel + bevacizumab once every 4 weeks. After 3 months, antihypertensive therapy (azilsartan 20 mg and amlodipine 5 mg) was initiated to treat her incremental blood pressure by the attending gynecologist. Detailed control of blood pressure was unclear because she did not measure home blood pressure. During the follow-up by the gynecologist, cardiologists were not consulted. After 7-months of chemotherapy including bevacizumab, the patient achieved a complete remission (Figure 1B). 

### 2.2. On Admission

At 2 months following the complete remission, the patient was admitted to our hospital complaining acute dyspnea and orthopnea. Her blood pressure was 140/89 mmHg and her heart rate was 113 bpm. There was a Levine 4/VI systolic apical murmur with evident gallop rhythm without peripheral edema. Serum N-terminal pro B-type natriuretic peptide was 2941 pg/mL. 

**Figure 2:**
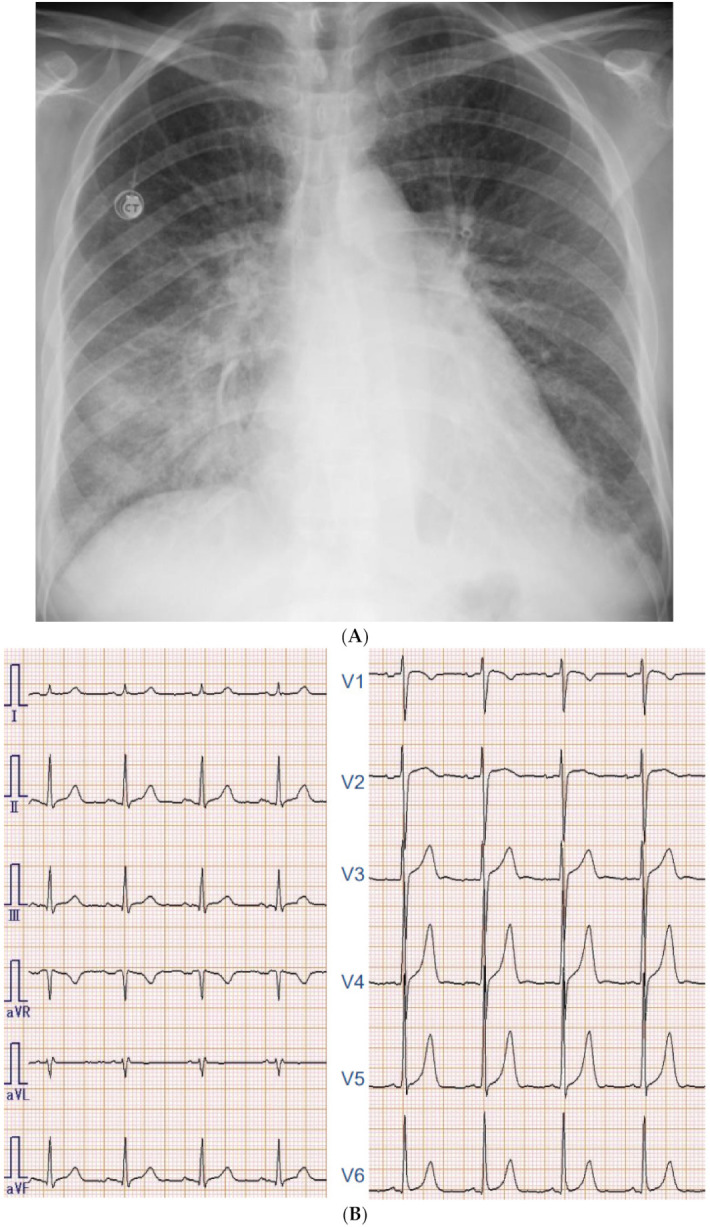

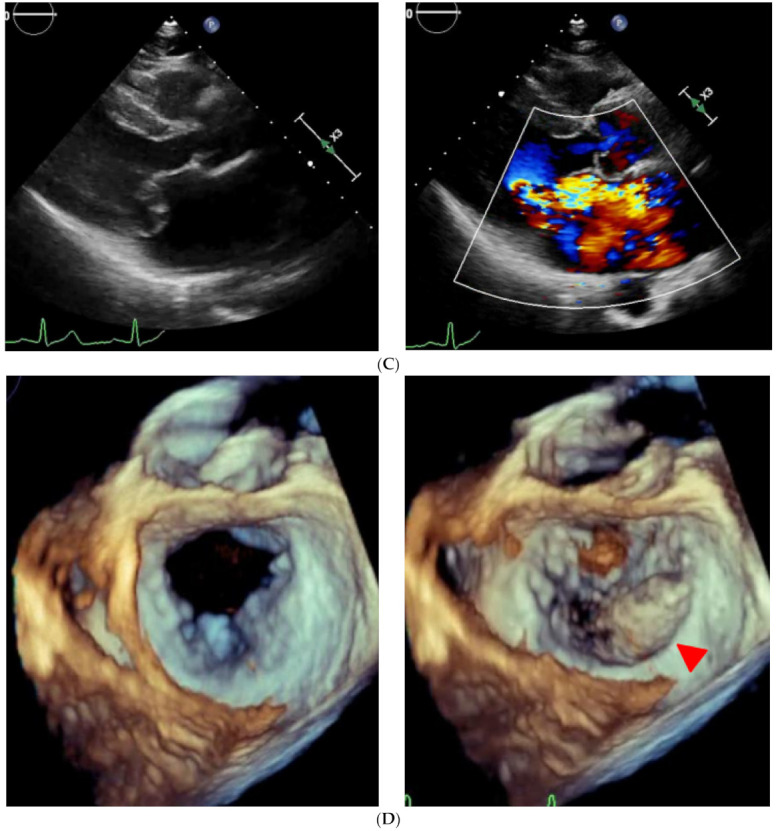
(**A**) Chest X-ray on admission showing slight cardiomegaly, pulmonary congestion, and the bilateral pleural effusions. (**B**) Electrocardiography on admission showing normal sinus rhythm and left ventricular hypertrophy. (**C**) Transthoracic echocardiogram showing severe mitral regurgitation due to billowing with posterior mitral valve prolapse. (**D**) Transesophageal echocardiography showing the prolapsed P2 and P3 segment with the degenerated and thickened leaflets.

Chest X-ray showed slight cardiomegaly, pulmonary congestion, and bilateral pleural effusions (Figure 2A). Electrocardiography showed normal sinus rhythm, high voltage without strain pattern, and longer ventricular activation time (Figure 2B). On transthoracic echocardiography, left ventricular end-systolic diameter was 34 mm with an ejection fraction of 72% calculated by modified Simpson method. There was no left ventricular hypertrophy. She had severe mitral regurgitation due to billowing and prolapse of the mitral valve (Figure 2C). Given these findings, we diagnosed acute heart failure due to severe degenerative mitral regurgitation.

### 2.3. In-Hospital Course

Given the diagnosis of acute heart failure with severe mitral regurgitation triggered by afterload mismatch, we initiated intravenous nitroglycerine and furosemide as well as non-invasive positive pressure ventilation. Transesophageal echocardiography found prolapsed P2 and P3 segments with degenerated and thickened leaflets of the mitral valve, which were compatible to Barlow’s disease (Figure 2D). Right heart catheterization showed a pulmonary artery pressure of 43/19/28 mmHg, pulmonary capillary wedge pressure of 31 mmHg, and cardiac index of 1.88 L/min/m^2^ using Fick’s method. 

The EURO SCORE (European System for Cardiac Operative Risk Evaluation) II was calculated as 0.93%. Given that she achieved complete remission and she had a degenerative mitral regurgitation instead of a functional one, the heart-valve team conference decided to perform surgical mitral valve repair rather than percutaneous repair. 

### 2.4. Post-Operative Course

There were no complications following the surgery with remaining trivial mitral regurgitation. One week after the surgery, left ventricular ejection fraction was 50% and serum N-terminal pro B-type natriuretic peptide decreased to 460 pg/mL. Following the surgery, the patient received enalapril 1.25 mg and amlodipine 5 mg and her systolic blood pressure was well controlled at around 120 mmHg.

Metastatic dissemination and local recurrence occurred at 1 month after the surgery (Figure 1C). Given the improved mitral regurgitation, preserved cardiac function, well-controlled blood pressure, and a considerable antitumor effect of bevacizumab without any alternatives, bevacizumab was re-administered at 3 months after the surgery. At 4 months of bevacizumab re-administration, she has not readmitted for heart failure.

## 3. Discussion

### 3.1. The Association between Bevacizumab and Heart Failure

Bevacizumab is a novel antibody that binds to vascular endothelial growth factor, which plays a key role in regulating angiogenesis in cancerous cells. Meta-analysis showed that patients treated with bevacizumab had a higher risk of developing cardiac and cerebral ischemia, bleeding complications, hypertension, and arterial and venous thromboembolism due to the endothelium damage and prothrombotic status [6,7,8,9].

The phenotypes, risk factors, and reversibility of heart failure are unknown. Given that the incidence of heart failure development during bevacizumab therapy is very small, some baseline cardiac comorbidities as risk factors might exist for the occurrence of drug-related heart failure [8,9]. Antitumor drugs sometimes affect heart valves, but in general it takes several years [2,10]. Given a relatively short duration of chemotherapy in this case, we assumed that our patient would have already had a subclinical degenerative mitral valve due to Barlow’s disease at baseline. In Barlow’s disease, the organization of the atrialis, spongiosam, and fibrosa in the mitral valve is disrupted, leading to more extensible leaflets and mitral prolapse [5]. Incremental afterload due to the adverse effect of bevacizumab would have worsened the mitral regurgitation, resulting in acute decompensated heart failure [2,11]. Further studies are warranted to investigate risk factors of heart failure occurrence during bevacizumab therapy [12,13].

### 3.2. Practical Implications of Cardio-Oncology Collaboration

With the growing problems, we need to address for (1) understanding the basic pathophysiologic mechanisms of cardiovascular disease in cancer patients, (2) identifying high-risk cancer patients who are likely to develop cardiovascular disease, and (3) translating these findings into personalized therapy to maximize oncologic effectiveness and minimize cardiotoxic potential [2,14].

Of note, our patient was not followed by the cardiologists before the index hospitalization. Including the high-risk cohort like our patient, those receiving bevacizumab should be consulted by cardiologists for careful follow-up to prevent cardiovascular events. Cross-disciplinary efforts are essential to reduce the mortality and improve the quality of life in cancer survivors.

## 4. Conclusions

The existence of mitral valve regurgitation, even when sub-clinical, might be a risk of worsening heart failure during bevacizumab therapy. Careful follow-up at onco-cardiology clinics might be recommended particularly for the high-risk cohort. Further studies are warranted to investigate the risk factors of worsening heart failure during bevacizumab therapy.

## Data Availability

Data are available from the corresponding author upon reasonable request.
